# Variations in the California Emergency Medical Services Response to Opioid Use Disorder

**DOI:** 10.5811/westjem.2019.12.45189

**Published:** 2020-04-16

**Authors:** Nancy K. Glober, Gene Hern, Owen McBride, Mary P. Mercer

**Affiliations:** * Indiana University, Department of Emergency Medicine, Indianapolis, Indiana; † Alameda Health System-Highland Hospital, Department of Emergency Medicine, Oakland, California; ‡ University of California, San Francisco, Department of Emergency Medicine, San Francisco, California

## Abstract

**Introduction:**

Opioids contributed to over 300,000 deaths in the United States in the past 10 years. Most research on drug use occurs in clinics or hospitals; few studies have evaluated the impact of opioid use on emergency medical services (EMS) or the EMS response to opioid use disorder (OUD). This study describes the perceived burden of disease, data collection, and interventions in California local EMS agencies (LEMSA).

**Methods:**

We surveyed medical directors of all 33 California LEMSAs with 25 multiple-choice and free-answer questions. Results were collected in RedCap and downloaded into Excel (Microsoft Corporation, Redmond WA). This study was exempt from review by the Alameda Health System - Highland Hospital Institutional Review Board.

**Results:**

Of the 33 California LEMSAs, 100% responded, all indicating that OUD significantly affects their patients. Most (91%) had specific protocols directing care of those patients and repeat naloxone dosing. After naloxone administration, none permitted release to law enforcement custody, 6% permitted patient refusal of care, and 45% directed base hospital contact for refusal of care. Few protocols directed screening or treatment of OUD or withdrawal symptoms. Regular data collection occurred in 76% of LEMSAs, with only 48% linking EMS data with hospital or coroner outcomes. In only 30% did the medical director oversee regular quality improvement meetings. Of respondents, 64% were aware of public health agency-based outreach programs and 42% were aware of emergency department BRIDGE programs (Medication Assisted Treatment and immediate referral). Only 9% oversaw naloxone kit distribution (all under the medical director), and 6% had EMS-based outreach programs. In almost all (94%), law enforcement officers carried naloxone and administered it anywhere from a few times a year to greater than 200 in one LEMSA.

**Conclusion:**

This study represents an important description of EMS medical directors’ approaches to the impact of OUD as well as trends in protocols and interventions to treat and prevent overdoses. Through this study, we can better understand the variable response to patients with OUD across California.

## INTRODUCTION

Drug overdoses have led to more than half a million deaths in the United States in the last 10 years, two-thirds of which were opioid-related.^1^ Opioid-related deaths increased fivefold from 1999 to 2016,^2^ and became the leading cause of accidental death in the US in 2015.^3^ While heroin and other illicit opioids contributed to the epidemic, prescription opioids constituted the bulk of the drugs leading to overdose.^4^–^6^ Although poorly characterized, the impact to the emergency medical services (EMS) system is significant. Naloxone administration was documented in nearly half a million EMS runs nationally from 2014 to 2016, as presented by the National EMS Information System (NEMSIS).^7^

In response, institutions and governments rolled out programs to curb the epidemic at every level of healthcare delivery. Broadly, the Centers for Disease Control and Prevention (CDC) recommended a five-pronged response: perform surveillance and research; empower consumers to make safe choices; build state, local, and tribal capacity; partner with public safety; and support providers, health systems, and payers.^8^ Specifically, the CDC recommended that states emphasize surveillance, policies, and funding to reduce prescription size and number, increase access to medication assisted therapy (MAT), and expand first responder access to naloxone.^8^ As a result, local, regional, and national governments implemented laws to reduce supply, monitor use, and enhance response to overdoses.^9^

Across the country, legal immunity has been granted to clinicians prescribing naloxone to third parties and bystanders aiding overdose patients in possession of illicit drugs.^10^,^11^ Naloxone is now widely available at pharmacies through physician standing orders.^10^,^11^ Community groups designed kits with naloxone and educational programs to teach laypersons how to correctly identify and treat opioid overdose.^12^–^14^ Many EMS medical directors expanded the scope of practice for naloxone administration to include law enforcement personnel and other non-paramedic first responders.^15^–^18^

Most research and data collection has focused on patients who use opioids in hospital and clinic settings. Little research explores the burden of disease and scope of response in the prehospital setting. In the state of California, oversight of EMS and coordination of care are accomplished through medical directors in 33 local emergency medical system agencies (LEMSA). According to the US Census Bureau, LEMSAs provide care to a population greater than 39 million that is spread across an area of nearly 156 thousand square miles, diverse in urbanicity and demographics. Each LEMSA regulates prehospital care with independent protocols that can vary widely from one region to the next. In this paper, we surveyed medical directors in all 33 LEMSAs within California to describe local approaches to opioid use disorder (OUD), access to data on patients who overdose from opioids, and community harm-reduction programs to prevent opioid overdoses.

## METHODS

In March 2019, we sent a survey via electronic mail to the medical directors of the 33 LEMSAs in California. One survey was to be completed for each LEMSA by either the medical director for that LEMSA or a representative. After six weeks, we sent reminder emails to medical directors who had not completed the survey. Incomplete or conflicting data was resolved with a follow-up phone call or electronic mail to the medical director.

We developed the survey by committee consensus. The survey highlighted three main outcomes: local perception of burden of OUD and protocols to respond to opioid overdoses; access to data on patients who overdosed from opioids; and community harm-reduction programs to prevent further opioid overdoses. Within those three areas, we asked 25 questions, in a combination of multiple-choice and free-answer formats (Appendix A). Results were collected in RedCap and downloaded into Excel (Microsoft Corporation, Redmond WA) for data analysis. We present a descriptive analysis of the results.

**Population Health Research Capsule**

What do we already know about this issue?

The Centers for Disease Control declared opioid overdoses to be a national health crisis.

What was the research question?

We surveyed the medical directors of all 33 California Local Emergency Medical Services (EMS) Agencies to define perception and response to opioid use disorder.

What was the major finding of the study?

While all Local EMS Agencies perceived the burden of opioid use disorder to be significant, few had built EMS-based outreach programs in response.

How does this improve population health?

We provide EMS agencies with information about variation in perception and response to opioid use disorder.

## RESULTS

### Local Perception And Protocols

**Figure 1:**
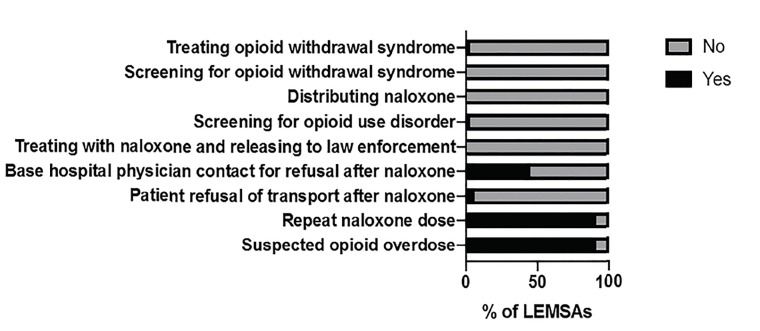
Percentage of emergency medical services agencies (LEMSA) that did or did not have protocols to understand and regulate a response to patients with opioid use disorder.

All 33 LEMSAs responded to the survey and all indicated that OUD significantly affects patients in their areas. Most (91%) had specific protocols directing care of those patients as well as directing repeat dosing of naloxone. When they existed, those protocols specified a range from one re-dose to unlimited, titrating to effect. Few LEMSAs had policies for patient refusal of care after naloxone administration, but nearly half had policies directing base hospital physician contact when a patient refused transport after naloxone administration. No LEMSA had a protocol to release a patient to law enforcement custody after naloxone administration, screen patients for withdrawal symptoms, or distribute naloxone to patients in the field. However, after base hospital physician contact was made, some LEMSAs (36%) permitted patients to be released to law enforcement custody after naloxone administration, and a few LEMSAs (9%) permitted treatment of patients with opioid withdrawal syndrome (Table 1 and Figure 1).

### Access to Data on Patients Who Overdose from Opioids

Of all LEMSAs, 76% collected data on opioid overdoses, but only 48% linked data with hospital and coroner outcomes. In only 30% did the medical director regularly oversee quality improvement (QI) meetings to review the data. The QI meetings varied from review of law enforcement naloxone administration to review of cases in which naloxone was administered, to review of presumed opioid overdoses by an epidemiologist. Of those with a regular QI process, 38% examined the geographic distribution of naloxone administration within their LEMSA.

### Community Programs to Prevent Opioid Overdoses

**Figure 2:**
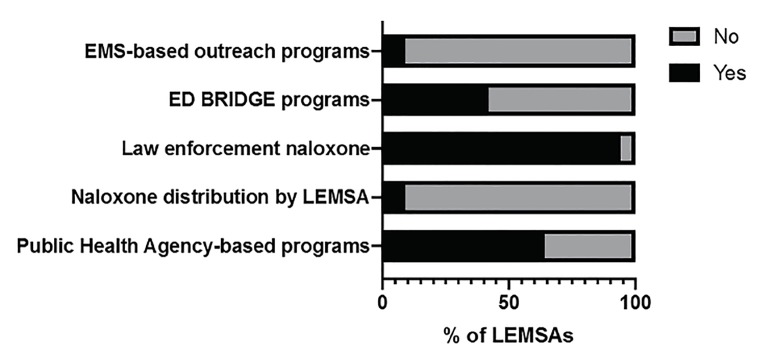
Percentage of emergency medical services agencies (LEMSA) that did or did not have programs to prevent opioid overdoses.

More than half of respondents were aware of public health agency-based outreach programs for harm reduction or emergency department (ED) BRIDGE programs – ED-based buprenorphine prescribing with rapid access to outpatient follow-up. When they existed, those programs varied widely and included law enforcement distribution of naloxone, public health outreach, multidisciplinary committees, ED referral to outpatient BRIDGE programs, and sheriff department distribution of naloxone to recently released inmates. In one LEMSA, the respondent replied that there was no such program, and in the rest (33%), the respondents indicated uncertainty as to whether such a program existed. Few LEMSAs oversaw naloxone kit distribution or had EMS-based outreach programs. In almost all (94%), law enforcement officers carried naloxone. Most started within the past two years, and one started four years ago. Use of law enforcement naloxone varied from a few uses a year to greater than 200 in one LEMSA (Table 2 and Figure 2).

## DISCUSSION

This study highlighted the variable EMS response to OUD across California. Prehospital administration of naloxone for patients with suspected opioid overdose has proven safe and effective for 30 years,^19^ and is now standard practice in the US.^20^ Our results suggest that California medical directors are uniformly aware of the impact of the opioid epidemic. Accordingly, LEMSAs widely adopted protocols for naloxone administration, re-dosing, and use by law enforcement, consistent with the national standard of practice.

Few LEMSAs implemented specific protocols allowing patients to refuse transport after naloxone. While emerging data from the prehospital setting indicates patients who sign out against medical advice following naloxone reversal have low mortality as a result of rebound toxicity,^21^–^24^ these studies were mostly limited to heroin and morphine overdose Little to no data exists on high-potency opioids (fentanyl and its analogs) or long-acting opioids. Current literature suggests an observation period of at least one hour following reversal of opioid overdose with naloxone in the ED.^25^,^26^ Given the rise in overdose deaths from high-potency opioids,^27^,^28^ some groups have recommended longer observation periods, up to six hours.^29^

The question of how to safely and ethically care for these patients is a complex one. Medically, further studies are needed to continue to evaluate the safety of patient release after naloxone administration in the setting of increasing use of high-potency synthetic opioids. Ethically, we must balance the medical prerogative of autonomy with that of non-malfeasance. We must allow those patients to determine their own medical care, while simultaneously evaluating whether or not administration of naloxone and subsequent release is consistent with the medical principle “first, do no harm.” Furthermore, non-transport of patients after naloxone administration could represent a lost opportunity to provide therapy and counseling in the ED.

Available data suggests that efforts aimed at increasing community access to naloxone decrease mortality from opioid overdose.^30^–^34^ In their guidelines on community management of opioid overdose, the World Health Organization issued a strong recommendation to distribute naloxone to individuals likely to witness an opioid overdose.^35^ Across the country, naloxone distribution programs are expanding in number and scope.^36^ EMS providers frequently encounter individuals at high risk for repeat overdose and individuals who are likely to witness an overdose.^20^,^37^ Despite this, only 9% of California LEMSAs oversee a naloxone distribution program. Further, only 64% were aware of public health outreach or harm reduction programs within their jurisdictions, and less than half of the LEMSA medical directors surveyed were aware of BRIDGE programs to connect patients with MAT centers. This demonstrates the disconnect between awareness and action and shows a potential growth area for LEMSAs to more fully address the public health burden. The development of EMS “leave behind” naloxone programs is an emerging strategy^38^–^40^ for stakeholders seeking to exploit this previously missed opportunity to intervene at the site of an overdose.

## LIMITATIONS

This study is limited in scope to examining EMS system protocols in only one state, California. Therefore, its findings may not be generalizable to trends or experiences in addressing opioid-related issues in other geographic settings. Additionally, as a survey based-study, the findings are limited to exploring approaches of EMS medical directors and protocols rather than examining outcome data for opioid-related EMS calls between LEMSAs. This study does not attempt to compare prehospital practices or outcomes with those described in hospital and clinic settings.

## CONCLUSION

Significant variation exists throughout the state in prehospital response to patients with opioid use disorder. The responses indicate an awareness of some harm-reduction principles for acute overdose (such as law enforcement naloxone), but little initiation of EMS-led programs such as naloxone distribution to the community sites or linkage with MAT programs. We hope that this study will prove useful to medical directors in California and throughout the US as they continue their efforts to respond appropriately and effectively to opioid use disorder.

## Supplementary Information



## Figures and Tables

**Table 1:** Percentage (number) of local emergency medical services agencies (LEMSA) with protocols to understand and regulate a response to patients with opioid use disorder.

Survey question	Affirmative response
Does your LEMSA have a specific protocol directing care for patients with suspected opioid overdose?	91% (30)
Does your LEMSA have a protocol for repeated dosing of naloxone?	91% (30)
Does your LEMSA have a specific protocol for patient refusal and release from care following administration of naloxone or suspected overdose?	6% (2)
Does your refusal policy require base hospital physician contact for refusals following naloxone administration?	45% (15)
Does your LEMSA have a specific protocol for treating a patient with naloxone and releasing the patient to law enforcement custody?	0% (0)
Does your LEMSA have a specific protocol for screening patients for opioid use disorder?	3% (1)
Does your LEMSA have a specific protocol for distributing a naloxone kit to patients?	0% (0)
Does your LEMSA have a specific protocol for screening patients for opioid withdrawal syndrome?	0% (0)
Does your LEMSA have a specific protocol for treating patients with opioid withdrawal syndrome?	3% (1)

**Table 2:** Percentage (number) of emergency medical services agencies (LEMSA) with programs to prevent opioid overdoses.

Survey question	Affirmative response
Are there any public health agency-based outreach or harm reduction programs for patients with opioid use disorder within the county(ies) served by your LEMSA?	64% (21)
Does your LEMSA oversee a naloxone kit distribution program?	9% (3)
Do law enforcement officers carry naloxone within the counties served by your LEMSA?	94% (31)
Are you aware of any emergency department BRIDGE programs within the counties served by your LEMSA? (Patients treated and discharged with buprenorphine-naloxone and referral)	42% (14)
Within your LEMSA, do you have any EMS-based outreach programs? (eg, paramedics distributing information to at-risk patients and family members, prehospital distribution of buprenorphine-naloxone, referrals by prehospital providers to treatment programs)	9% (3)
