# Supplementary material for: Variations in the California Emergency Medical Services Response to Opioid Use Disorder
Source: West J Emerg Med. 2020 Apr 16;21(3):671–6. doi: 10.5811/westjem.2019.12.45189 (PMC7234694; doi:10.5811/westjem.2019.12.45189)
Supplement: Supplementary file 1 [file wjem-21-671-s001.docx]

**Appendix A**

This survey seeks to gather information regarding the impact of opioid use disorder (OUD) and EMS policies throughout California pertaining to OUD.

It is divided into three sections:

1. Perception of the impact of opioid drug use in the prehospital setting and current protocols for care of patients with an opioid overdose.

2.  Quality improvement efforts and data pertaining to opioid overdose data in the prehospital setting.

3.  Programs in place or that being developed to combat OUD.

We direct this survey to the medical directors of each LEMSA in California (one survey per LEMSA), or their designated representative. Please complete this survey only once.   As with past surveys of this group, responses will be anonymous. Findings will be shared in an aggregated fashion for the purposes of regional policy discussion as well as research. Your LEMSAs participation is voluntary, and your consent is implied by your response to this survey.  Should your LEMSA have a unique protocol or best-practice that you would like to have shared in a non-anonymous fashion during reporting of our findings, you will have the opportunity to indicate this on the survey.

We would appreciate any additional comments, elaborations or questions to the survey administrators (listed below) in response to this survey.

Nancy Glober MD ([nancy.glober@stanford.edu](mailto:nancy.glober@stanford.edu))

H. Gene Hern MD ([emergentt@gmail.com](mailto:emergentt@gmail.com))

Mary Mercer MD, MPH ([Mary.mercer@ucsf.edu](mailto:Mary.mercer@ucsf.edu))

## **topic 1: medical director perception and basic protocols**

Please select the LEMSA in which you are responding for.

1. Does opioid abuse or misuse significantly impact your LEMSA?
   1. It is not a problem.
   2. It is a problem in general, but less so in our LEMSA.
   3. It is a big problem in our LEMSA, but we do not have EMS programs to monitor or intervene.
   4. It is a big problem, and we have EMS programs built to monitor and intervene in an effort to prevent future overdoses.
2. Does your LEMSA have a specific protocol directing care for patients with suspected opioid overdose?
   1. Yes
   2. No
3. Does your LEMSA have a protocol for repeated dosing of naloxone?
   1. Yes
   2. No
4. If you answered yes to 3, how many repeat doses are directed?
   1. ______
   2. Not applicable (I answered “No” to question 3)
5. Does your LEMSA have a specific protocol for treating a patient with naloxone and releasing the patient from care?
   1. Yes, it specifically addresses opioid overdose patients
   2. No, but paramedics have the option to call a base station for further direction
   3. We have a protocol for patients who wish to be released after treatment, but it does not specifically address patients who were given naloxone for presumed opioid overdose
6. Does your LEMSA have a specific protocol for treating a patient with naloxone and releasing patient to law enforcement custody?
   1. Yes
   2. No
   3. No, but it may happen after calling a base station
7. Does your LEMSA have a specific protocol for screening patients for opioid use disorder?
   1. Yes
   2. No
   3. No, but it may happen after calling a base station.
8. Does your LEMSA have a specific protocol for distributing a naloxone kit to patients?
   1. Yes
   2. No
   3. No, but it may happen after calling a base station.
9. Does your LEMSA have a specific protocol for screening patients for opioid withdrawal syndrome?
   1. Yes
   2. No
   3. No, but it may happen after calling a base station.
10. Does your LEMSA have a specific protocol for treating patients with opioid withdrawal syndrome?
    1. Yes
    2. No
    3. No, but it may happen after calling a base station.

## **topic 2: Access to data on patients who overdosed from opioids**

1. Does your system collect and report data on suspected opioid overdoses?
   1. Yes
   2. No
2. Does your LEMSA have a regular QI process for suspected overdoses that you, as medical director, oversee?
   1. Yes
   2. No
3. If your LEMSA has a regular QI process, please describe below (report monthly, annually, quarterly case review, etc)
4. If your LEMSA has a regular QI process, do you review your data as it differs by region within your LEMSA?
   1. Yes
   2. No
5. Does your system give you access to hospital or coroner case outcomes that you can link to a specific ambulance run?
   1. Yes
   2. No

## **topic 3: community programs to prevent opioid overdoses**

1. In your LEMSA, are there naloxone kit distribution programs?
   1. Yes
   2. No
2. If the answer to question 1 is yes, how is naloxone distributed? (how much, which areas, how is it funded, who distributes it – paramedics, EMTs, community health workers?)
3. In your LEMSA, do law enforcement officers carry naloxone?
   1. Yes
   2. No
4. If so, when did they start (year)?
5. If law enforcement officers in your LEMSA carry naloxone, how many times has it been used (per year)? (put I don’t know if you do not review it)
6. Are you aware of any emergency department bridge programs within your LEMSA? (Patients treated and discharged with buprenorphine-naloxone and referral)
   1. Yes
   2. No
7. Within your LEMSA, do you have any EMS-based outreach programs? (for example, paramedics distributing information to at-risk patients and family members, prehospital distribution of suboxone, referrals by prehospital providers to treatment programs)
   1. Yes
   2. No
   3. Don’t know
8. If you answered “Yes” to 7, please circle all known below.
   1. Screening for at risk settings (houses with left over prescriptions, high users of regular narcs
   2. Distribution of information substance use disorders
   3. Distribution of information on how to access treatment programs
   4. Direct Referral to treatment programs
   5. Prehospital Distribution of naloxone kits
   6. Prehospital Distribution of buprenorphine-naloxone
   7. Referral for public health outreach programs (community health visits to recent overdose patients
   8. Other: please describe below
9. Within your LEMSA, do you have any Public Health Agency-based outreach programs?
   1. Yes
   2. No
   3. Don’t know
10. If you answered “Yes” to 9, please describe below.
